# Microcephalic Osteodysplastic Primordial Dwarfism in Two Siblings Living in Sindh, Pakistan: A Case Report

**DOI:** 10.7759/cureus.10258

**Published:** 2020-09-05

**Authors:** Shahzeen Saifullah Khan, Muhammad Hasan Shahab

**Affiliations:** 1 Pediatrics, Dow Medical College/Dr. Ruth K. M. Pfau, Civil Hospital, Karachi, PAK

**Keywords:** primordial dwarfism, seckle syndrome, majewski osteodysplastic primordial dwarfism, microcephaly, mopd 2

## Abstract

Microcephalic osteodysplastic primordial dwarfism type 2 (MOPD2) is a rare autosomal recessive disorder that presents as a myriad of skeletal abnormalities collectively termed as osteodysplasia, which have their onset during intrauterine life with the fetus exhibiting intrauterine growth restriction. Affected individuals also tend to have a very small head size that is more than three standard deviations (SD) below the mean for a population termed microcephalic. The growth problems progress postnatally, causing stunted growth or short stature. In this report, we present the case of two siblings: a 15-year-old unvaccinated female weighing 8 kg (case one), and a seven-year-old unvaccinated female weighing 6 kg (case two), who presented to the Department of Pediatrics, Unit 2 at the Civil Hospital in Karachi, Pakistan, complaining of short stature since birth.

## Introduction

Microcephalic osteodysplastic primordial dwarfism type 2 (MOPD2), is a rare condition, characterized by small head size, short stature, and skeletal abnormalities. The disease has an autosomal recessive inheritance pattern and can present with a variety of additional manifestations involving multiple organ systems, such as vascular anomalies (especially in the brain), abnormal skin pigmentation, and subglottic stenosis. The growth problems associated with MOPD2 have their onset well before birth, with affected individuals demonstrating intrauterine growth retardation (IUGR). The growth abnormalities continue postnatally, with the final height in adulthood reaching no more than 20-40 inches. This condition is thought to be the result of a mutation in the PCNT gene encoding a protein called pericentrin, which anchors various centrosomal proteins and other protein complexes during mitosis, contributing to a wide variety of pathological manifestations due to abnormal cellular division [1].

Considering the significant overlap between different forms of primordial dwarfism and the unknown prevalence of the condition itself, quantifying the frequency of MOPD2 is arduous. As such, the literature and records available on this disease are not abundant and, therefore, its dynamics have yet to be fully elucidated. For Pakistan in particular, there is a scarcity of published literature on this condition. One report documenting the presence of symptoms of hyperandrogenism in a patient with known MOPD2 by Kiran et al. published in 2017 is the only other piece of evidence that sheds any light on the condition [2]. This report therefore shall highlight the prospects of MOPD2 in the above-mentioned cases in light of previously documented literature.

The diagnosis of this condition is complex, and hence it is based mainly on a combination of a thorough medical history, physical examination, radiological tests, laboratory tests, and, most importantly, genetic testing involving the pericentrin gene (where possible). This condition can be confused with other forms of primordial dwarfisms and conditions with moderate to severe IUGR, such as Seckel syndrome, MOPD1, MOPD3, Russell-Silver syndrome, Antley-Bixler syndrome, Fanconi syndrome, and Meier-Gorlin syndrome [3]. However, based on the evidence available, the main features that ultimately predominate in MOPD2 and distinguish it from the aforementioned conditions are as follows: bony dysplasia, unusual skin pigmentation, and, according to Perry et al. (2013), cerebrovascular disease at some point in life [4].

## Case presentation

Two siblings, who were residents of Tharparkar, Sindh, one a 15-year-old unvaccinated female weighing 8 kg, and the other a seven-year-old unvaccinated female weighing 6 kg, presented to the outpatient department (OPD) for evaluation of short stature since birth. According to the mother, both her daughters had been delivered very small at birth and had remained small for their ages thereafter. She also complained that her children were unable to socialize and raised concerns that they might be mentally retarded as they depended completely on their parents for performing their daily activities.

The mother had not visited any hospital to receive antenatal care, and she had not received any supplementation or vaccination during either pregnancy. Both pregnancies had resulted in uneventful, spontaneous vaginal deliveries at full term, with her daughters having very low birth weights, which the mother could not recall nor had any record of. There was no history of delayed cry, apnea, cyanosis, or any other complications postnatally. Both girls had been breastfed till two years of age, with weaning beginning at four months of age. The developmental history provided by the mother was suggestive of delayed milestones; case one had begun neck-holding at eight months of age, sitting without support at 16 months of age, standing at three years, walking at four years, and had said her first words at 4.5 years of age. Case two had also exhibited significant but comparatively less severe delays, with neck-holding having begun at seven months of age, sitting without support at 12 months of age, standing at two years, walking at 3.5 years, and speaking at four years of age.

Both children were unvaccinated. There was no history of either child having suffered recurrent infections, nor was there any history of blood transfusions, seizure disorders, or allergies. Case one had sleep disturbances and a reduced appetite while bowel and bladder functions were normal. Case two, however, did not suffer from any sleep disturbances, had a normal appetite, and also had normal bowel and bladder control.

There were no complaints of the children having any nausea, vomiting, diarrhea, altered bowel habits, or abdominal distension, which would have otherwise prompted suspicion of an underlying metabolic disorder. There was also no history of headache, altered levels of consciousness, dizziness, or fainting spells. The mother reported that the elder child (case one) had experienced multiple episodes of fits, which were described as sudden stiffening of the body followed by jerky movements of the arms and legs with up-rolling of eyes, each lasting less than five minutes, before arriving at the hospital. Her past history was significant for such fits occurring intermittently without any attributable cause. The child was not taking any anti-epileptic medication. Case two exhibited no such symptoms. Systemic reviews involving the cardiorespiratory systems were also not significant. The mother complained that case one often complained of backaches and experienced difficulty walking. There was no history of trauma. Case one had achieved menarche within six months of turning 15. Case two had not yet achieved menarche and the rest of the systemic review involving the genitourinary system was unremarkable. The mother reported both her children having hypo- and hyper-pigmented patches all over their trunk. There were no complaints of earache, ear discharge, tinnitus, or vertigo.

The two children and their mother came from a family of seven individuals, products of a consanguineous marriage, with no history of short stature, low birth weight, or mental illness in the rest of the children. The family was socioeconomically poor, the father being a laborer by profession, and the only breadwinner for the house.

Case one details

On examination, case one was apparently short-statured and mildly emaciated but well-oriented regarding time, place, and people. The child was vitally stable. Her height was 87 cm, more than 8 standard deviations (SD) below normal and microcephalic, as suggested by an occipitofrontal circumference (OFC) of 38 cm, which was more than 11 SD below normal. The child was also grossly under-weight, weighing only 8 kgs, which was less than the 5th centile for her age, with a mid-upper arm circumference (MUAC) measuring 11 cm, and an arm span measuring 89 cm. Additionally, she had an upper-to-lower segment ratio equal to 1.

The girl had a bird-headed, elongated face, with a beaked nose, absent earlobes, and a squeaky high-pitched voice. She had sparse scalp hair and multiple hypo- and hyper-pigmented macules present all over her trunk. She also had small, abnormally shaped teeth. There was a restricted range of motion in her right elbow joint. The examination of her back revealed mild thoracic scoliosis, which was confirmed by Adam's forward bend test. A scoliometer could not be used as it was not available in the hospital. There was no leg-length discrepancy. Examinations of the abdomen, cardiovascular, respiratory, and central nervous systems were unremarkable. Assessment of visual acuity and refractive error revealed the presence of mild hyperopia of +0.25 in both eyes. Ear, nose, and throat (ENT) evaluations were normal. The child had normal female genitalia at Tanner stage 2.

Routine hematological investigations were carried out at presentation, which showed mild anemia with hemoglobin (Hb) of 11.0 gm/dl (lab normal reference range: 12.0-15.5 gm/dl). Blood electrolytes, urea, creatinine, and total protein levels were within normal limits. A fasting lipid profile was carried out, which revealed a normal level of low-density lipoprotein (LDL) cholesterol, 118 mg/dl (lab reference range: 60-130 mg/dl), and slightly low levels of high-density lipoprotein (HDL) cholesterol, 38 mg/dl (normal reference range: >45mg/dl). Fasting blood sugar was elevated at 128 mg/dl (normal range: 70-99 mg/dl) with an HbA1c of 6.5% (normal reference range: 4-5.6%). The thyroid profile was also normal. An ultrasound of the abdomen and pelvis along with echocardiography was performed, which also revealed no significant findings.

**Figure 1:**
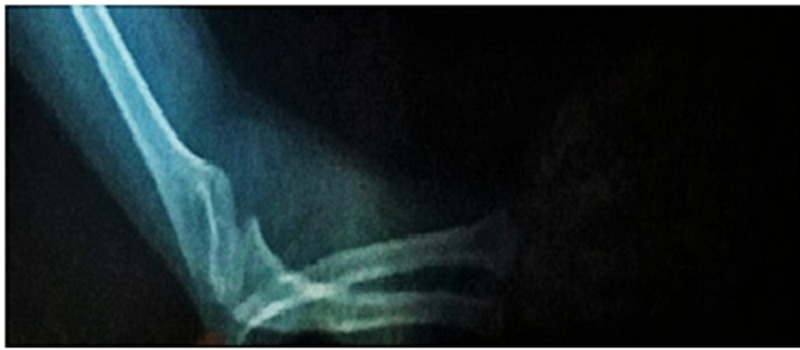
Case one: evidence of posterior dislocation of the radial head as seen in the lateral view of the right elbow X-ray Image quality turned out to be poor as the child was agitated and irritable, making radiographic imaging extremely difficult

A lateral view of the right elbow on the X-ray was significant for the posterior dislocation of the radial head, as shown in Figure 1.

**Figure 2:**
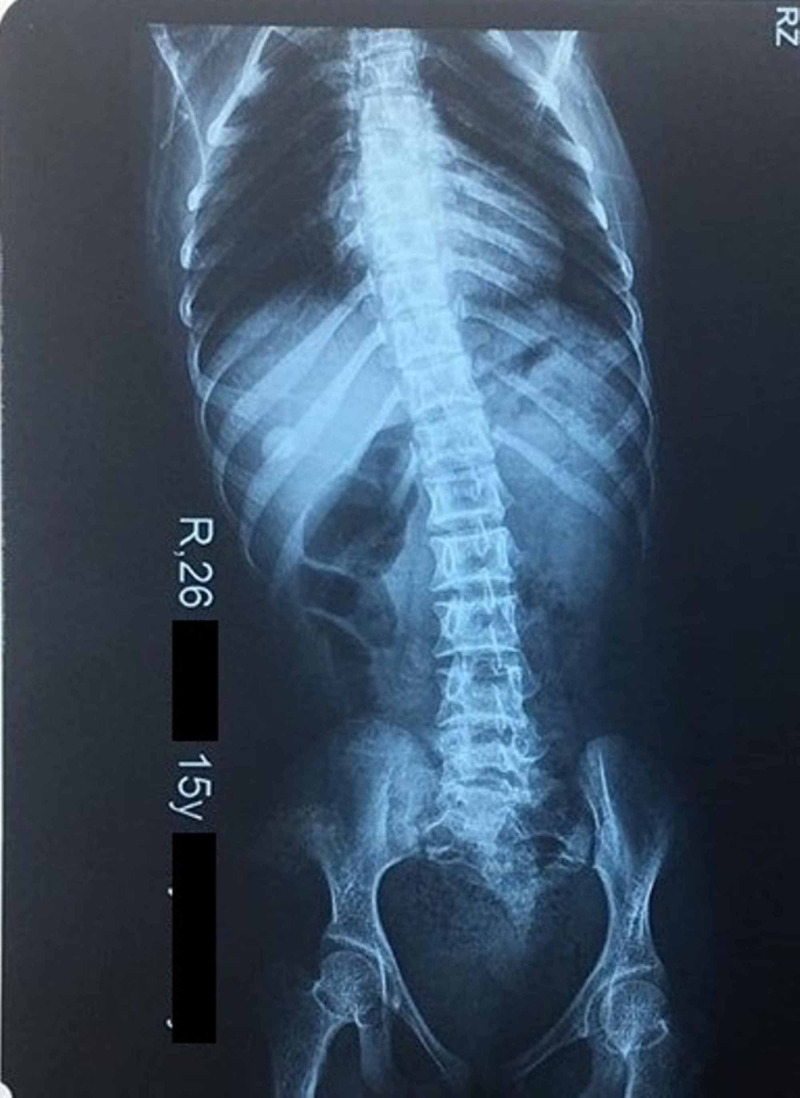
Case one: thoracic scoliosis as evident on the scoliosis X-ray

A scoliosis X-ray revealed mild scoliosis as shown in Figure 2. No other major skeletal dysplastic findings could be appreciated.

**Figure 3:**
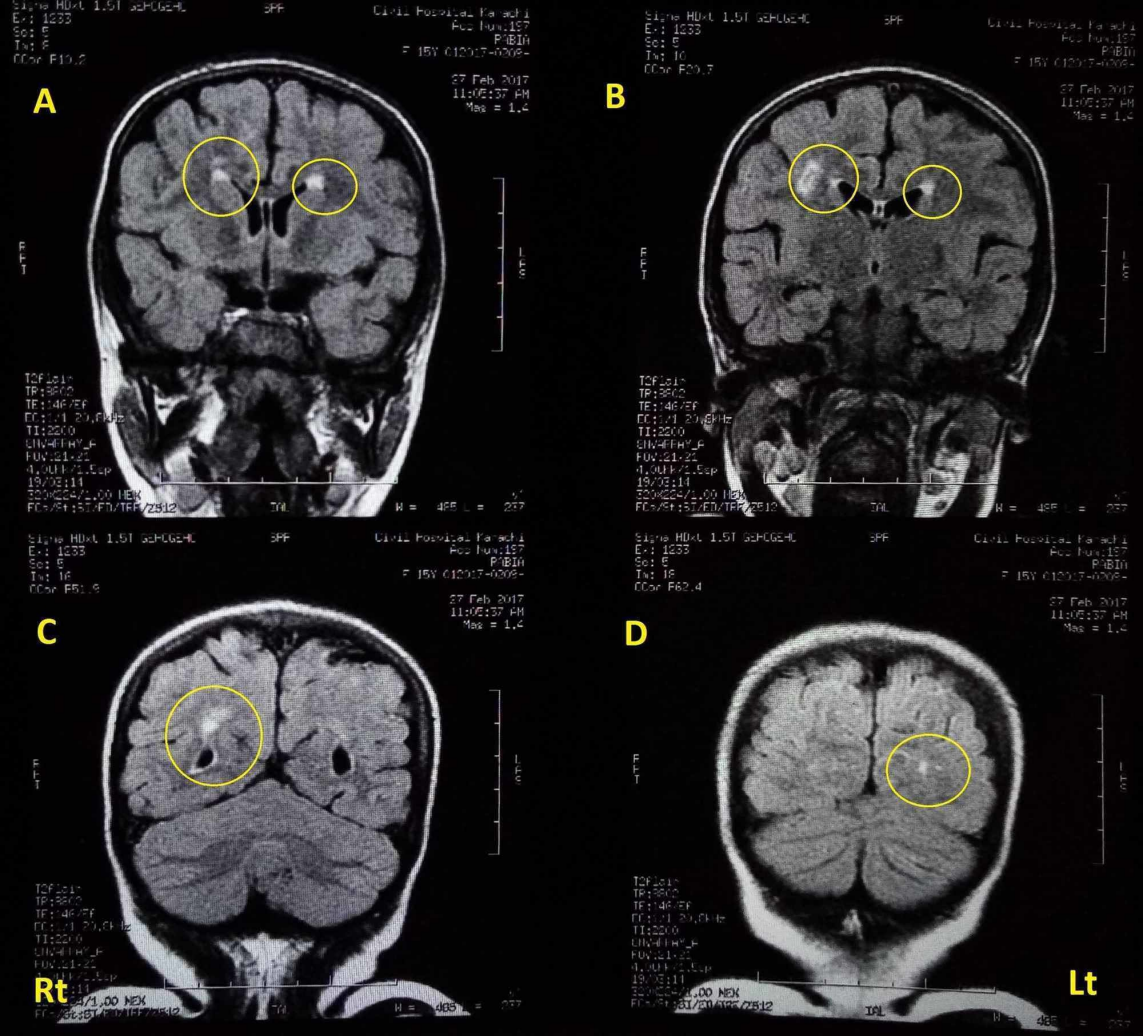
Case one: MRI Brain showing changes consistent with occlusive arteriopathy, with attenuation of bilateral middle and posterior cerebral circulation Areas of involvement, encircled in the image, can be prominently seen in the MRI FLAIR sequence. Yellow circles indicate areas of old paraventricular ischemia/infarction MRI: magnetic resonance imaging; FLAIR: fluid-attenuated inversion recovery; Rt: anatomical right side of the brain; Lt: anatomical left side of the brain

Due to the unavailability of a magnetic resonance angiogram (MRA) machine, the limited resources available at the government-funded hospital, and the poor socioeconomic status of the patient's family, an MRA could not be performed to further confirm the findings of the MRI. An MRI of the brain revealed changes consistent with occlusive arteriopathy with bilateral old paraventricular lesions, hyperintense on the fluid-attenuated inversion recovery (FLAIR) MRI sequences. The lesions in the FLAIR MRI sequence, encircled in Figure 3, were strongly suggestive of preferential middle and posterior cerebral arterial involvement. 

A psychiatric opinion was also taken, according to which case one had moderate mental retardation as per the Stanford-Binet IQ classification.

Case two details

On examination, case two was a seven-year-old girl of short stature, thin build, and dysmorphic facies, who was well-oriented with time, place, and people. She was vitally stable. Examination of her eyes revealed conjunctival pallor suggestive of anemia. Jaundice, cyanosis, clubbing, and dehydration were not present. The thyroid gland along with lymph nodes was not palpable. Case two also had a bird-headed elongated face with a beaked nose, small earlobes, and squeaky high-pitched voice. She had small teeth with dental caries. She also had thin and sparse scalp hair. There were multiple hypo- and hyper-pigmented macules present all over her trunk.

Anthropometric examination revealed the following: an OFC of 37 cm, more than 9 SD below the mean for her age; height of 69 cm, more than 10 SD below the mean for her age; a weight of 6.2 kg, less than the 5th percentile for her age; a mid-upper arm circumference of 11.5 cm; an upper-to-lower-segment ratio of 1.1; and an arm span measuring 65 cm.

On inspection of the lower limbs, the patient had small patellar prominences present laterally and prominent femoral condyles with a hollow sulcus indicative of the patellar surface of the femur. Assessment of visual acuity and refractive error revealed the presence of mild hyperopia (+0.25). ENT examination was normal.

Routine hematological investigations were carried out at presentation, which revealed mild anemia with a Hb level of 10.0 gm/dl (normal range: 12-15.5 gm/dl). Blood biochemistry [electrolytes, blood urea nitrogen (BUN), creatinine, and nutritional profile] was within normal limits. The fasting lipid profile was normal. However, fasting blood sugar was elevated at 130 mg/dl (normal range: 70 to 99 mg/dl) with an HbA1c of 7.0% (normal reference range: 4.0-5.6%).

**Figure 4:**
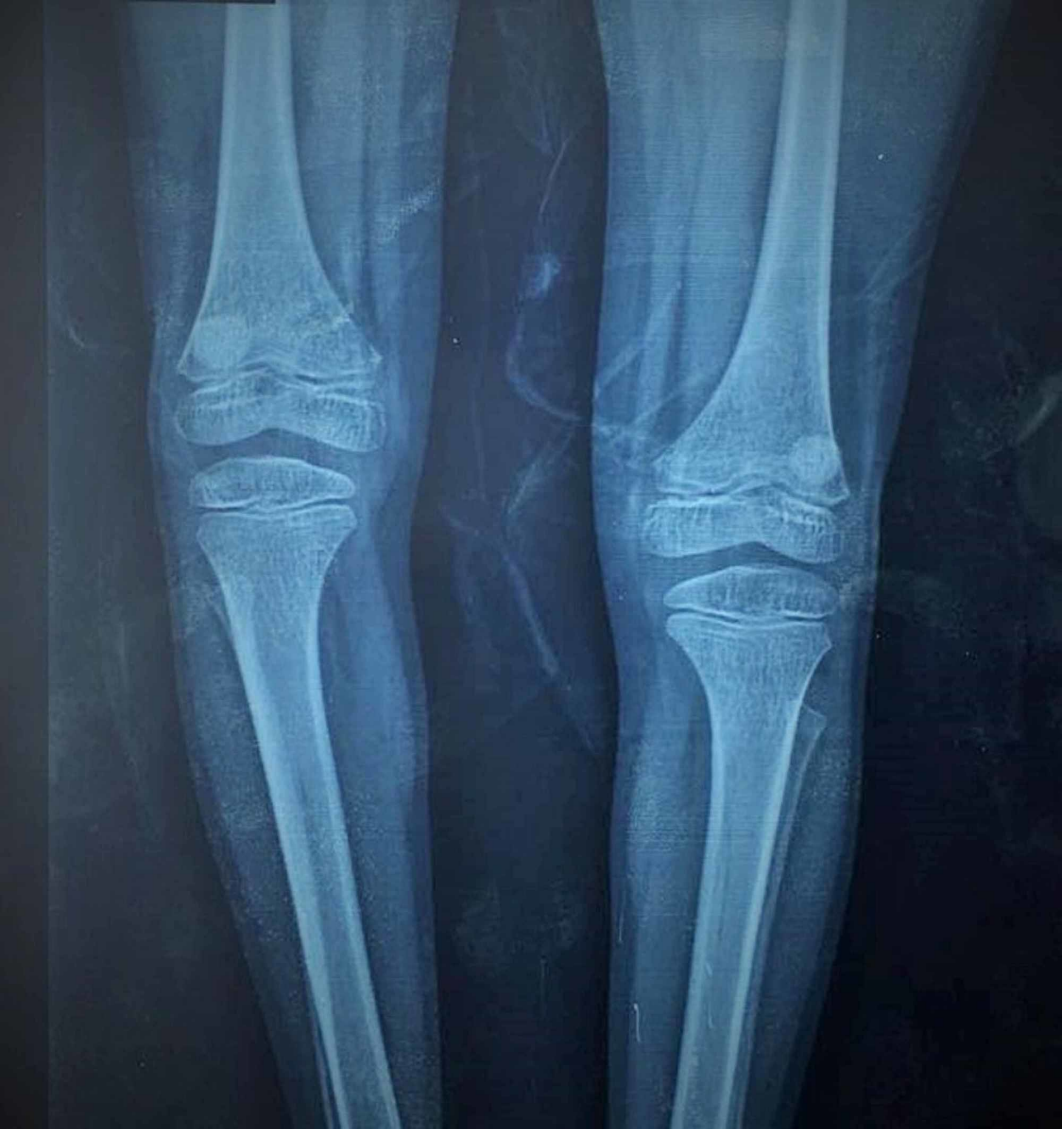
Case two: bilateral hypoplastic and subluxated patellas with varus deformity as evident on the X-ray The image indicates a very rare presentation of MOPD2, with bilateral hypoplastic patellae MOPD2: microcephalic osteodysplastic primordial dwarfism type 2

The thyroid profile was normal. An ultrasound of the abdomen and pelvis along with echocardiography was performed, which also revealed no significant findings. X-rays of both knee joints, as shown in Figure 4, revealed bilateral subluxation of hypoplastic patellae, with a varus deformity.

A psychiatric opinion was sought, according to which case two had mild mental illness as per the Stanford-Binet IQ classification. An MRI was not carried out as there were no significant central nervous system findings or complaints.

## Discussion

On a gross outlook, both our cases shared the typical features of MOPD2: osteodysplasia as exhibited by the presence of skeletal abnormalities in the form of dysmorphic facies, disproportionately shortened limbs, overall growth retardation, mental retardation, and microcephaly. According to Hall et al. (2004), who reviewed 58 cases (27 from literature and 31 previously unreported), the hallmark features consistently found were severe IUGR, severe postnatal growth retardation, abnormalities consistent with osteodysplasia described above as well as a myriad of other findings - "progressive loose-jointedness with occasional dislocation or subluxation of the knees, radial heads, and hips; unusual facial features including a prominent nose, eyes that appear prominent in infancy and early childhood, ears that are proportionate, mildly dysplastic, and usually missing the lobule; a high squeaky voice; abnormally small, and often dysplastic or missing, dentition; a pleasant, outgoing, sociable personality; and autosomal recessive inheritance. Hyperopia, scoliosis, unusual pigmentation, and truncal obesity often develop with time. Some individuals seem to have increased susceptibility to infections. There was variability between affected individuals even within the same family" [5].

Case one exhibited all the typical facial features of MOPD2, with dysplastic ear lobules, sparse hair, small teeth, and a squeaky voice. The child had multiple areas of hypo- and hyper-pigmented skin on her body, with thoracolumbar scoliosis and posterior dislocation of the radial head of her right arm, as evident on physical examination as well as the X-rays.

Case 2 shared most of these features with her sibling except for scoliosis and radial head dislocation. However, an unusual and interesting finding was the presence of bilateral subluxated hypoplastic patellas with a concurrent varus deformity. As mentioned previously, while dislocation and subluxation of the knee and hip joints are an oft-occurring skeletal abnormality in cases of MOPD2, there have been no reported cases of MOPD2 with coexisting patellar hypoplasia to date. The child had no unusual nail changes, which would have otherwise pointed towards a diagnosis of nail-patella syndrome, making this the first documented case of MOPD2 with congenital patellar hypoplasia.

Of note, 5-29% of cases of MOPD are known to suffer from seizures as mentioned in the Human Phenotype Ontology database. Case one had a past history of occurrence of seizures, described as generalized tonic and clonic. There were, however, no significant findings or localizing signs on examination of the central nervous system. The seizures could be attributable to vascular changes present in the brain as shown in the MRI.

Children with MOPD2 have significant intrauterine and postnatal growth failure. According to Fukuzawa et al., who reported autopsy findings in a Japanese girl exhibiting clinical and radiological features distinctive of MOPD2, impairment of chondrocyte formation and differentiation was found to be the underlying pathogenetic mechanism contributing to the development of MOPD2 [6]. In our case, since the mother belonged to a poor socioeconomic class, with no history of formal education, and living in a rural area of Pakistan where access to healthcare facilities and general amenities was limited, there was no documented record of her receiving antenatal care, routine ultrasound scans to monitor intrauterine growth, or checkups or postnatal care, which would have otherwise added to the validity of the diagnosis of MOPD2. According to the mother, both her children had been very small at birth, but their weights had not been measured or documented.

Developmental delays were seen in both of our cases although with slight variation between the two children. According to Hall et al., these delays are exacerbated by multiple factors, in particular feeding problems, familial IQ, and breathing difficulties in infants. However, there were no such complaints reported by the mother at birth. A compounding factor in our cases may be that the children were born to uneducated and illiterate parents. Depending on these variables, developmental milestones may either be normal or can be delayed by almost 50%. Most individuals, however, ultimately can communicate, ambulate, and carry out daily tasks by varying degrees of efficiency but are not able to live independently due to the vast array of disabilities. While both cases were able to communicate, they relied on their parents completely for performing their normal daily activities.

Case one (elder sibling) showed mild scoliotic changes, which was found by Hall et al. (2004) to be more common in females suffering from MOPD2, especially in late childhood and early puberty. She also showed changes in her MRI consistent with occlusive arteriopathy, as exhibited by bilateral old paraventricular lesions (hyperintense on FLAIR imaging) and evidence of bilateral middle and posterior cerebral arterial attenuation. According to a review of 147 published cases by Perry et al. (2013), such changes were observed in 32% (almost one third) of MOPD2 cases and with occlusive arteriopathies occurring at an earlier age compared to aneurysmal disease [4].

So far, there are no studies in the literature documenting an association of MOPD2 with deranged fasting lipid profiles as evident in case one. The abnormality could be accounted for by an unhealthy diet considering the poor socioeconomic status of the family.

According to a retrospective data from 26 individuals with MOPD2, in a study conducted by Bober et al., when the participants reached skeletal maturity, the height, weight, and OFC were -10.3, -14.3, and -8.5 SD below the population mean, respectively. The growth failure was accounted for by mutations in the pericentrin gene, responsible for microtubule assembly and cell division [7]. Both our cases had severe microcephaly, growth stunting, and both were significantly under-weight for their age as evident from their anthropometric measurements mentioned previously. While some participants of the study by Bober et al. were subject to treatment with growth hormones, none exhibited any significant improvement in their final stature [7].

The pericentrin gene has a possible role in regulating blood glucose levels and, hence, early onset of type 2 diabetes in affected individuals. Jurczyk et al. have observed the role of pericentrin in regulating the docking of insulin secretory vesicles in mice pancreatic beta cells. It was observed that normal animals had sufficient quantities of pericentrin in their beta cells. RNA mediating silencing of pericentrin resulted in abnormal, dysregulated insulin hypersecretion from secretory vesicles. Mice that were transplanted with pericentrin-depleted beta cells could not regulate their glucose levels after a glucose challenge [8]. Since pericentrin genetic testing is not available in most hospitals in Pakistan due to the high expense of the test and the very limited resources in most government-funded tertiary care hospitals, a definitive cause for the deranged sugar levels with the other myriad of findings could not be elucidated. Pericentrin mutation could, however, be a cause in our cases. 

MOPD2 has an autosomal recessive inheritance pattern, which was evident in both our cases who were products of a consanguineous marriage; the parents, however, exhibited no features of MOPD2, and nor was there any history of dwarfism in other members of the extended family. Verloes et al. and Al-Ghazali et al. have reported cases of MOPD2 born in families with a history of consanguinity [9].

## Conclusions

MOPD2 is a rare autosomal recessive disorder with a wide variety of manifestations such as growth retardation, bony dysplasias, and cerebrovascular disease. The exact incidence rate of the condition in our country is unknown owing to its rarity and the lack of documentation. This warrants raising sufficient awareness and knowledge among clinicians to be able to recognize its clinical manifestations and act promptly to maximize the quality of life for the patients. Parents of children afflicted by the disorder have to be counseled about how to care for their children, who may be prone to unintentional injury and isolation from the society owing to their short stature. Children presenting with dwarfism should be examined and worked up, a high index of suspicion for MOPD2 and its associations should be present in the physician's mind, and findings should be thoroughly documented, so that the incidence of this disorder can be determined and appropriate actions can be taken to manage patients with primordial dwarfism.

## References

[1] (2020). Microcephalic osteodysplastic primordial dwarfism type II. https://ghr.nlm.nih.gov/condition/microcephalic-osteodysplastic-primordial-dwarfism-type-ii#definition.

[2] Kiran Z, Furqan S, Farooq S, Rashid MO (2017). Microcephalic (Majewski) osteodysplastic primordial dwarfism type ii with severe hyperandrogenism. Endocr Pract.

[3] (2020). Microcephalic osteodysplastic primordial dwarfism type 2. https://rarediseases.info.nih.gov/diseases/9844/microcephalic-osteodysplastic-primordial-dwarfism-type-2.

[4] Perry LD, Robertson F, Ganesan V (2013). Screening for cerebrovascular disease in microcephalic osteodysplastic primordial dwarfism type II (MOPD II): an evidence-based proposal. Pediatr Neurol.

[5] Hall JG, Flora C, Scott CI Jr, Pauli RM, Tanaka KI (2004). Majewski osteodysplastic primordial dwarfism type II (MOPD II): natural history and clinical findings. Am J Med Genet A.

[6] Fukuzawa R, Sato S, Sullivan MJ, Nishimura G, Hasegawa T, Matsuo N (2002). Autopsy case of microcephalic osteodysplastic primordial "dwarfism" type II. Am J Med Genet.

[7] Bober MB, Niiler T, Duker AL (2012). Growth in individuals with Majewski osteodysplastic primordial dwarfism type II caused by pericentrin mutations. Am J Med Genet A.

[8] Jurczyk A, Pino SC, O'Sullivan-Murphy B (2010). A novel role for the centrosomal protein, pericentrin, in regulation of insulin secretory vesicle docking in mouse pancreatic β-cells. PLoS One.

[9] (2020). Microcephalic osteodysplastic primordial dwarfism, type II; MOPD2. https://omim.org/entry/210720#26.

